# Supplementary material for: EPINEST, an agent-based model to simulate epidemic dynamics in large-scale poultry production and distribution networks
Source: PLoS Comput Biol. 2024 Feb 21;20(2):e1011375. doi: 10.1371/journal.pcbi.1011375 (PMC10911595; doi:10.1371/journal.pcbi.1011375)
Supplement: S1 Text — File containing further details about data analysis as well as model simulation and initialisation. (PDF) [file pcbi.1011375.s001.pdf]

Supplementary Methods for the manuscript "EPINEST, an agent-based model to simulate epidemic dynamics in large-scale poultry production and distribution networks"

Contents

1 Data analysis **1**
1.1 Farmers . . . 1
1.2 Middlemen . . . 2
1.3 Vendors . . . 4

2 Actor dynamics **7**
2.1 Farms . . . 7
2.2 Middlemen . . . 8
2.3 Markets . . . 11
2.4 Vendors . . . 11

3 PDN setup **12**
3.1 Geography setup . . . 13
3.2 Farm generation . . . 13
3.3 Market setup . . . 15
3.4 Vendor setup . . . 15
3.5 Middlemen setup . . . 16

4 Simulating epidemic spread **17**
4.1 Within-farm transmission . . . 17
4.2 Inter-farm transmission . . . 19
4.3 External introductions . . . 20

1 Data analysis

1.1 Farmers

Questionnaire data

Data were generated through a cross-sectional study that collected information about farming practices from 100 distinct farms in Chattogram division,

Bangladesh [1]. Available data include farm sizes, the number of production cycles completed in one year, as well as the numbers of traders and transactions involved in clearing individual batches (Fig A). Here we focus on the 47 farms that reported trading broilers and raising a single batch at a time in a single shed. It should be noted, however, that while the questionnaire aimed to uncover farmers' practices over one year, it is not possible to know whether farmers raised at most one batch at a time for the entire period as their answers reflect their situation during the interview.

Farm/batch size. We modelled batch sizes S_F by assuming a truncated negative binomial distribution. More in detail, we assumed:

$$S_F \sim \mathcal{NB}(p_{S_F}, n_{S_F}) \text{ if } S_F \in [S_F^{min}, S_F^{max}] \,, \tag{1}$$

where S_F^{min}, S_F^{max} are the minimum and maximum observed batch sizes. For simplicity, we estimated parameters p_{S_F}, n_{S_F} by maximum likelihood without accounting for truncation and verified a posteriori that the resulting bias was small. Note that for this type of farms, batch size is the same as farm size, since they do not rear multiple batches at the same time. We find that this distribution provided a visually better fit than a Poisson distribution, which fails to account for the overdispersion in observed farm sizes (see Fig AQ).

Replenishment time. We modelled replenishment time $\tau_{replenish}$ by assuming a shifted negative binomial distribution, i.e.:

$$\tau_{replenish} - 1 \sim \mathcal{NB}(p_{\tau_{replenish}}, n_{\tau_{replenish}}) \,. \tag{2}$$

This distribution provided a visually better fit to empirical data than a Poisson distribution (see Fig AR).

1.2 Middlemen

Individual middlemen properties

We used data from questionnaires to inform middlemen's trading patterns. Consistently with the main manuscript, we restrict our analysis to broiler data only [2].

Number of chickens bought daily. We divided the total number of chickens bought during the entire study period by each middlemen with the number of days in which they purchased any poultry. We used these raw values to construct a discrete distribution from which to sample middlemen batch sizes S_{MM} in the ABM.

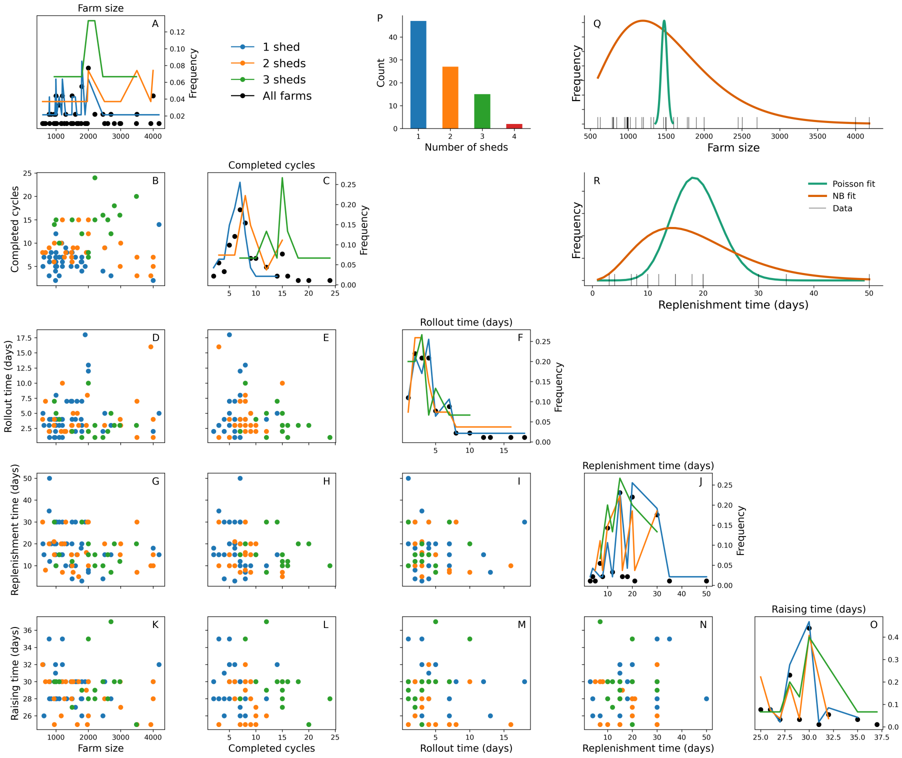

Figure A: **Farm statistics from survey data and statistical fits.** (A-O) Univariate distributions (diagonal) and pairwise scatter plots (off-diagonal) for several farm statistics, coloured by the number of available sheds. Shown quantities include farm size (A), completed production cycles per year (C), trading rollout duration (F), time to replenish the farm (J) and raising time (O). We did not show farms with 4 sheds, as there were only two (see panel P showing frequencies of sheds per farm). (Q) Poisson (green) and truncated negative binomial (orange) fits to batch size data (black ticks). (R) Poisson (green) and shifted negative binomial (orange) fits to replenishment time data (black ticks). In both cases, a negative binomial distribution provides a better fit than Poisson to underlying data. Analyses in panels Q,R were restricted to farms with a single shed.

Number of markets visited daily. As a first step, we evaluated the number of working days for each middleman by combining sale frequencies of different breeds. This was straightforward for middlemen that sold chickens every day during the study or sold a single chicken breed. One middleman declared selling chickens 4 times in a week; in this case, we set the number of working days to 4. We then obtained the mean number of daily market visits k^{daily} by dividing the total number of market visits made by each middleman during the study

period with the corresponding number of working days. Note that some values k^{daily} are not integer numbers due to the previous calculation. Finally, we used maximum likelihood to fit a geometric distribution with parameter p_{k_m} to data $\{k_i^{daily}\}$, with i ranging from 1 to the total number of middlemen interviewed. More precisely, the maximum likelihood estimate for p_{k_m} is given by the inverse of the sample mean of data $\{k_i^{daily}\}$. This formula works also when some values k_i^{daily} are not integer numbers (Fig B).

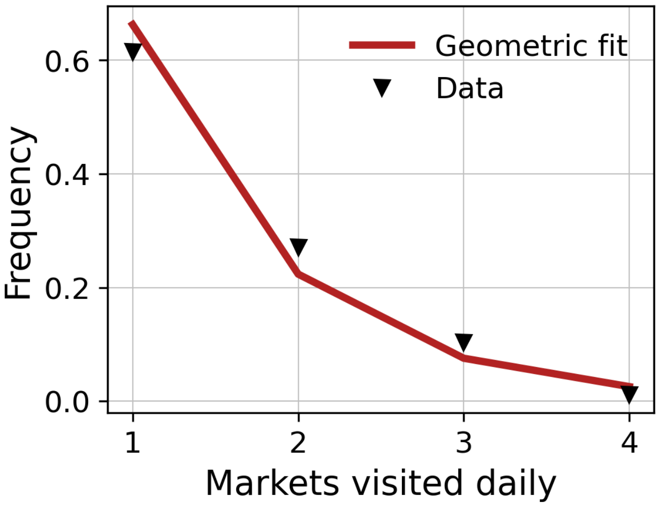

Figure B: **Distribution of daily market visits.** Fitted (line) and observed (markers) frequencies of markets visited daily. The fitted distribution is geometric with parameter p estimated using maximum likelihood. Note that some data points were not integers and were therefore rounded to the nearest integer in this plot. The fitted distribution corresponds to black markers in Fig 2E, but is here shown up to value 4, i.e. the maximum observed count.

1.3 Vendors

Individual vendor properties

We used data from questionnaires to inform vendors' trading patterns. Unless otherwise stated, we repeat every analysis for retailers and wholesalers.

Number of chickens bought daily. We first reconstructed the number of chickens bought by a single vendor over the survey period by adding counts of sold and unsold chickens. Then, we divided this number by the number of days during which the same vendor bought any chickens, yielding an estimate of the number of chickens bought daily. We used these raw values to construct

a discrete distribution from which to sample vendor batch sizes S_V in the ABM (Fig CA).

Surplus chickens First, we estimated the probability p_{empty} to sell the entire batch of chickens before a vendor buys another batch. This was computed as the ratio between the number of days with any unsold chickens and the number of days with a purchase.

Second, we modelled the counts u_{batch} of unsold chickens per batch, conditional on batch size S_V and on the event that the batch is not fully emptied (which happens with probability $1 - p_{empty}$). We consider a negative binomial distribution with mean $\mu = \rho_{unsold} S_V$ and variance $\mu \cdot (1 + n_{unsold}^{-1} \cdot \mu)$, where ρ_{unsold} is the proportion of chickens that remain unsold before purchasing the next batch and n_{unsold} affects overdispersion compared to a Poisson distribution. Because our data are aggregated over a week period, we model the total count of unsold chickens u_{total} rather than u_{batch}. Because the sum of negative binomial random variables still follows a negative binomial distribution, u_{total} has the same distribution as u_{batch}, with S_V multiplied by the number of days with any unsold chickens (as we are conditioning on having a surplus). Aggregating data from wholesalers and retailers yields maximum likelihood estimates $\rho_{unsold}^{MLE} = 0.13$ and $n_{unsold}^{MLE} = 3.4$ (Fig CB and CC), which are used throughout the main manuscript. For completeness, we also estimate the same parameters for wholesalers and retailers separately, who amount to 55 and 376 data points, respectively. We find that wholesalers display, on average and conditional on at least one unsold chicken, less surplus chickens than retailers ($\rho_{unsold}^{MLE,W} = 0.06$ vs $\rho_{unsold}^{MLE,R} = 0.13$), while the amount of overdispersion is roughly the same ($n_{unsold}^{MLE,W} = 3.4$ and $n_{unsold}^{MLE,R} = 4.2$). Note that we assume p_{empty} to differ between retailers and wholesalers. It should also be added that in the context of questionnaire data, a surplus of chickens is defined in relation to consecutive purchases, which could be multiple days apart, by the same vendor. In the ABM, however, we conflate these parameters with surplus between consecutive days, as vendors tend to buy new chickens every day. The resulting discrepancy should not be large since 76% of interviewed vendors purchased (broiler) chickens every day, and 93% during at least 6 days in a week. Finally, note that during simulations, daily surplus is also subject to the constrain that it can not be larger than batch size.

Prioritising unsold chickens. We estimate the probability of a vendor prioritising the sale of previously unsold chickens as the proportion of vendors declaring to do so in our data.

Transaction networks and inter-tier fluxes

Here we detail a procedure to estimate $p_W^{(L)}$ and $p_R^{(L)}$ for each market tier $L = 0, 1, \ldots, L_{max}$. Parameters $p_{W,R}^{(L)}$ represent the proportions of chickens sold respectively to wholesalers and retailers in tier L, while $1 - p_W^{(L)} - p_R^{(L)}$ is

the proportion of chickens that wholesalers in tier $L-1$ ($L > 1$) sell to end-point consumers.

The tier $L = 0$ corresponds to vendors that buy directly from middlemen. Note that $p_W^{(0)} + p_R^{(0)} = 1$ since middlemen are not allowed to sell chickens to end-point consumers. Wholesalers in tier L sell to wholesalers and retailers in tier $L+1$ according to probabilities $p_W^{(L+1)}$ and $p_R^{(L+1)}$, respectively. Note that the last tier L_{max} must consist of retailers only and hence $p_W^{(L_{max})} = 0$.

Here we outline a simplified estimation procedure that yields parameters that are common to all markets. We start with reconstructed transaction networks. These networks are DAGs consisting of multiple transactions, weighted by the number g of chickens involved, between different node types. Nodes can be either middlemen (MM) or vendors, the latter being further classified into either wholesalers (W) or retailers (R). In addition, MM and W nodes are further classified according to their 'depth' within a transaction chain. This latter characterization is somewhat reminiscent of our tiered structure, but a direct mapping is not possible at this stage yet. In particular, in order to infer relevant parameters from reconstructed transaction chains, we must take care of transactions involving middlemen that purchase chickens directly from wholesalers and sell them to other vendors. As our ABM does not allow middlemen to buy from vendors, we replace these 'forbidden' transactions found in reconstructed networks, with sets of 'allowed' transactions. Let V_{MM}^* denote the set of such middlemen. As a first step, we remove all transactions involving poultry farms, except those involving any middlemen $k \in V_{MM}^*$. For each remaining farm i, we replace middlemen $k \in V_{MM}^*$ with a new label k^* so that each transaction $i \to k$, $k \in V_{MM}^*$ becomes $i \to k^*$. The label k^* is just a placeholder representing a fictitious middleman. We then remove transactions of the type $i \to k$ and $k \to j$, where i, j are not farms and $k \in V_{MM}^*$, and replace them with viable transactions of the type $i \to j$. Thus, edges (i, k) and (k, j) representing chickens moving from i to j through $k \in V_{MM}^*$ are replaced by an edge (i, j) with weight $g_{i,j} = g_{i,k} g_{k,j} / g_k$, where $g_k = \sum_l g_{k,l}$ is the total out-weight of k. Finally, we remove transactions involving only middlemen and merge any duplicated edges together.

We can now analyse the resulting graph G. Let V_{MM} represent the set of middlemen nodes, i.e. the roots of the DAG. Note that V_{MM} excludes nodes from V_{MM}^*. We associate middleman i with a weight $g_i^{(0)}$ representing the number of chickens it 'injects' into markets. For each middleman node $i \in V_{MM}$, we then perform the following calculation:

- We enumerate all transaction chains departing from node i and ending with a leaf node. The latter might be a W or an R node. We stress that these chains represent observed transactions, not the output of our ABM.

- For each such chain we count the number of mark-ups, distinguishing between R and W nodes. Each chain may involve any number of W nodes and eventually a terminal R node. For example, a chain of the type $W \to W \to R$ consists of two wholesalers mark-ups before landing

into a retailer. A chain of the type $W \to W$ consists of two wholesalers mark-ups, with the terminal wholesaler selling directly to end-consumers. Finally, we assign a weight to the current chain by multiplying $g_i^{(0)}$ by the proportion of chickens that reach end-consumers through this chain. The latter is easily computed by multiplying together the proportions of chickens flowing through each edge in the chain. It is easy to see that the weights of chains emanating from node i must add up to $g_i^{(0)}$.

By enumerating all chains constructed in this way, we can easily determine the maximum number of tiers as the length of the longest chain in G. For example, if the longest chain is of the type $W \to W \to R$, then we need 3 tiers and $L = 0, 1, L_{max} = 2$.

Once we have computed the weights for every chain in G, we can finally obtain $p_W^{(L)}$ and $p_R^{(L)}$ by collapsing all chains according to their length and the type of terminal node. The idea is to compute a running sum $g_G(L, T)$ that counts how many chickens end up in the hands of actors of type T, where T is either W, R or C (end-point consumer) in tier L. Please note that we are deliberately over-counting chickens according to how many mark-ups they make. Let us illustrate this procedure by considering a generic chain with weight g_c, $n_W >= 0$ wholesaler and $n_R = 0, 1$ retailer mark-ups. The total length of the chain is $n_c = n_W + n_R$. For each $L = 0, \ldots, n_W - 1$ we increase the running sum $g_G(L, W)$ by g_c. If the last node is of type W, we increase the running sum $g_G(n_W, C)$ by g_c; else, the last node is of type R and we increase the running sum $g_G(n_W, R)$ by g_c.

Finally, $p_W^{(L)} = g_G(L, W)/[g_G(L, W) + g_G(L, R) + g_G(L, C)]$ and $p_R^{(L)} = g_G(L, R)/[g_G(L, W) + g_G(L, R) + g_G(L, C)]$. Note that $g_G(0, C) = 0$ if we ignore transactions involving MM selling to end-point consumers.

Note that we can include more reconstructed transaction networks in the same analysis: we compute $g_G(L, T)\ \forall G$ and then create a consensus quantity $g_{consensus}(L, T) = \sum_G g_G(L, T)$, which we can finally use to compute parameters of interest.

2 Actor dynamics

This section describes in detail different action and tasks for each actor. Further details on actor instantiation can be found in the section dedicated to PDN setup.

2.1 Farms

At any time, a farm is either empty or raising chickens. We consider farms raising a single batch of chickens at a time, meaning that individual production cycles do not overlap. Chickens from the same batch are introduced at the same time as day-old chicks and are offered for sale as soon as they reach an

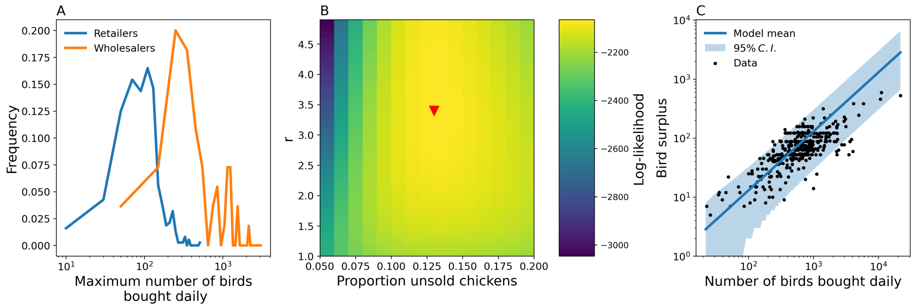

Figure C: **Vendors' parameters analysis.** (A) Raw distributions of numbers of birds bought daily for R (blue) and W (orange). (B) Log-likelihood function for negative binomial fit to surplus counts. Fitted parameters include the average proportion on unsold chickens ρ_{unsold} and the shape parameter n_{unsold}. The maximum likelihood solution $(\rho_{unsold}^{MLE}, n_{unsold}^{MLE})$ is indicated with a red marker. (C) Expected surplus $\rho_{unsold}^{MLE} \cdot S$ (line) as a function of batch size S, together with 95% C.I. (shaded area) calculated assuming a negative binomial distribution $\mathcal{NB}(\rho_{unsold}^{MLE} S, n_{unsold}^{MLE})$ for surplus. Broiler data are shown as well (black dots).

appropriate age τ_{raise}. A production cycle ends when all chickens in a farm are sold.

Farm replenishment. After completing a cycle, a farm remains empty for a random time $\tau_{replenish}$, sampled from a (shifted) negative binomial distribution. After $\tau_{replenish}$ time steps, a farm recruits a new batch of day-old chicks.

Offering birds for sale. After raising a batch for τ_{raise} days, a farm is ready to sell chickens. These are offered for sale progressively over a minimum of $\tau_{rollout}$ days; more precisely, a fraction $1/\tau_{rollout}$ of chickens in a batch become available for sale every day during the first $\tau_{rollout}$ days. By day $\tau_{rollout}$, all chickens at the farm can be sold to middlemen. Note that a farm could sell chickens for a longer period of time if there are not enough middlemen to trade with. In the case where any chickens remain unsold after $\tau_{rollout}^{(max)}$ days, the corresponding farm is emptied automatically.

2.2 Middlemen

The daily routine of a middleman consists of buying and collecting chickens from farms in order to sell them to vendors operating in markets. A single middleman is able to source chickens from multiple farms, potentially from distinct regions, in order to fill up their cargo.

Updating scouted regions. Each middleman purchases chickens from farms located in a subset $\mathcal{A}$ of neighboring areas, whose number we denote with $n_{scout} = |\mathcal{A}|$. With daily frequency, each middleman may move and change his/her catchment area with probability P_{move}. In that case, a middleman updates $\mathcal{A}$ by choosing n_{scout} new regions as follows: first, a focal region a is sampled at random with propensity proportional to the number of chickens being currently offered for sale in that region. In the case no farm is currently trading, a is chosen fully at random. Second, $n_{scout} - 1$ new regions are chosen at random among those neighboring a. More precisely, we say that a region a' neighbors a if their centroids are less than 80 km far apart. If a has less than $n_{scout} - 1$ neighbors, new regions are chosen among the areas neighboring with a's direct neighbors. The choice of a 80 km radius is arbitrary, but it guarantees that middlemen do not cover too long distances in a single day. Note also that according to our algorithm, middlemen are drawn preferentially to areas with the largest offer of chickens, which reduces the odds of farms not being able to sell their chickens.

Buying birds from farms. The main purpose of mobile traders is to collect chickens from farms and deliver them to LBMs. With daily frequency, our algorithm allocates amounts of chickens to be moved from any trading farm to any market via middlemen. No chicken is collected at this stage yet. Our simulator resolves allocations one middleman at a time in random order and under the following constraints:

1. Middleman j can hold at most S_{MM} chickens.
2. Middleman j can source chickens from farms located in any area $a \in \mathcal{A}$.
3. Middleman j sells chickens to a number of vendors from exactly k_m markets.
4. Destination markets must be chosen in a way the preserves the overall proportion of chickens leaving region a and entering market l, $f_{a,l}$.

As a consequence of the last two requirements, each middleman keeps track of which market each chicken is scheduled to be moved to. The first step consists in sampling markets with probabilities $\{q_l\}_{l=1,\dots,N_M}$:

$$q_l = \frac{\sum_{a \in \mathcal{A}} \tilde{O}_a f_{a,l}}{\sum_{a \in \mathcal{A}} \tilde{O}_a}\ , \tag{3}$$

where $\tilde{O}_a$ is the number of birds currently offered for sale by farms in region a. We sample markets with replacement until we get a set $\mathcal{M}$ comprising k_m distinct markets, or after 1000 draws, in which case $|\mathcal{M}| < k_m$. Let t_l be the number of times market $l \in \mathcal{M}$ was selected during sampling, and let $t = \sum_l t_l$ be the total number of draws. Focusing on market l with $t_l > 0$, we construct the distribution $f_{a|l}$:

$$f_{a|l} = \begin{cases} \dfrac{\tilde{O}_a f_{a,l}}{\sum_{a \in \mathcal{A}} \tilde{O}_a f_{a,l}} \text{ if } a \in \mathcal{A} \\ \\ 0 \text{ otherwise} \end{cases} . \tag{4}$$

We then sample tokens $t_{l,a} \sim Multinomial(t_l, \{f_{a|l}\})$ such that $\sum_{a \in \mathcal{A}} t_{l,a} = t_l$. Given the tokens $t_{l,a}$, middleman j contacts farms in each region $a \in \mathcal{A}$, securing up to $\left\lfloor \tilde{S}_{MM} t_{l,a} / t \right\rfloor$ birds destined to market l. Here, $\tilde{S}_{MM} \leq S_{MM}$ denotes the maximum number of birds that can be purchased during the current day. $\tilde{S}_{MM}$ is smaller than S_{MM} if middleman j is already carrying birds for any reason, e.g. if unsold from previous days (this is usually rare).

Middlemen contact farms in an area a sequentially, starting with those trading for the longest amount of time. Transactions follow a greedy heuristics: if middleman j has to collect s_a birds from region a, they will attempt to buy as many birds as possible from individual farms to meet such quota. In case middleman j fails to secure the required number of birds from region a, we allow j to contact any previously visited farms to meet the original quota. Additional chickens obtained in this way are directed to market l with probability:

$$\begin{cases} \dfrac{f_{a,l}}{\sum_{l' \in \mathcal{M}} f_{a,l'}} \text{ if } l \in \mathcal{M} \\ \\ 0 \text{ otherwise} \end{cases} . \tag{5}$$

Finally, it should be noted that $\tilde{O}_a$ decreases as chickens are allocated progressively to middlemen; in other words, availability of chickens decreases as we iterate over middlemen.

Bird collection. Middlemen start collecting birds as soon as the allocation step described in the previous section is terminated. At this point, middleman j is already aware of the farms to be visited and the amounts of birds to pick up from each of them. In simulations, middleman j can visit any of these farms in random order at any point in time before the next market opening. This is to ensure that j collects all of their chickens before bringing them to the market.

Selling birds to vendors. Each day, middleman j visits markets $l \in \mathcal{M}$ sequentially. There, j sells chickens to available vendors until all s_l carried chickens scheduled for delivery to market l have been sold. These chickens can be sold only to vendors in the first market tier ($L = 0$). A number $s_l(W) \sim Binomial(s_l, p_W^{(0)})$ are allocated for sale to wholesalers in this tier. The remaining $s_l - s_l(W)$ chickens, plus any of the $s_l(W)$ chickens that could not be sold to wholesalers (e.g. because of limited buying capacity) are directed to retailers. Any unsold chickens remain in a middleman's stock and are offered

for sale in the same market on the following day. Please note that this event is expected to occur rarely in simulations.

2.3 Markets

Markets can be either open or closed: all markets open at T_{open} and close at T_{close} every day. Markets contain chickens only during opening hours.

2.4 Vendors

Vendors trade chickens within and between markets. There are two types of vendors: retailers, who sell chickens to end-point consumers only, and wholesalers, who can also sell chickens to other vendors. Vendors are organized in tiers: vendors in tier $L > 0$ buy chickens from wholesalers in tier $L - 1$, while vendors in tier $L = 0$ buy chickens directly from middlemen. Vendors first purchase chickens in market l and then move to another market l', or stay in l, where they can sell chickens to other vendors and/or end-point consumers.

Buying birds. All vendors seek to buy as many chickens as possible during any transaction with middlemen and/or other vendors, compatibly with their own capacity and daily quota S_V. Note that a vendor may hold more than S_V chickens at a time due to the presence of surplus chickens. Nonetheless, the same vendor can not hold more chickens than the maximum carrying capacity $C_V > S_V$.

Inter-market movements At T_{open}, vendor k moves to market l to purchase chickens. Eventually, k stays in the same market or moves to a second location l' to trade. Chickens move alongside their owner. Note that k's purchase and trade markets l,l' do not change in time: k will always purchase chickens in l and commute to l' (if $l \neq l'$) or stay in l (if $l = l'$) in a given PDN realisation.

All vendor movements are resolved at T_{open} and are therefore instantaneous: vendors move to market l, purchase chickens and eventually change market during the same time step. Note that vendors in tier L must move after those in tier $L - 1$.

Wholesaling. Let us consider a wholesaler k operating in tier L. After purchasing chickens, k carries n_{tot} chickens, including also older chickens that remained unsold from previous days. In the current day, k sells $n_{sale} = n_{tot} - n_{unsold}$ chickens, where n_{unsold} denotes the number of unsold chickens. n_{unsold} is a random variable whose sampling procedure is described in sections below. Proportions $p_W^{(L+1)}$ and $p_R^{(L+1)}$ of these n_{sale} are then directed to wholesalers and retailers in tier $L + 1$, respectively. The remaining chickens are sold to end-point consumers (retailing).

It is possible that k sells less chickens to $L+1$ tier wholesalers than planned. In this case, any surplus chickens are redirected to retailers. Similarly, any

chickens that would remain unsold after the wholesaling phase are redirected to end-point consumers.

Please note that wholesaling is instantaneous, occurring toe-to-toe with inter-market movements as vendors switch from purchasing to trading chickens.

Retailing. Both wholesalers and retailers can sell chickens to end-point customers. While wholesaling is instantaneous, retailing rolls out over market opening hours, i.e. between T_{open} and T_{close}. More in detail, vendors can sell chickens to end-point consumers between $T_{open} + 1$ and $T_{close} - 1$; this means that chickens spend at least one time step at the market.

The number of chickens sold in any time step over this period is stochastic, but uniformly distributed. More in detail, we assume that the number of chickens sold by vendor k during time step t is given by:

$$min\{n_{avail}(t), round\,(u + X(t))\}\ , \tag{6}$$

where X is a Poisson random variate with mean $n_{avail}(t)/\delta t$, with $n_{avail}(t)$ and δt denoting the number of chickens destined to retail still in stock and the time left until market closes, respectively. u is a random draw from an uniform distribution on the unit interval whose role is to fix rounding issues, while $round(.)$ guarantees a meaningful result by rounding $u + X$ to the nearest integer. Finally, we require that k sells all of the remaining chickens that had been scheduled for retail, right before market closure, i.e. at $t = T_{close} - 1$.

Surplus chickens. As explained above, vendor k may retain a random number n_{unsold} of chickens each day, which are then offered for sale the day after. The surplus n_{unsold} is computed as follows: first, vendor k sells the entire stock with probability p_{empty}, in which case $n_{unsold} = 0$. Alternatively, n_{unsold} is sampled from a negative binomial distribution with mean $\rho_{unsold} \cdot n_{tot}$, with the constraint $n_{unsold} \leq n_{tot}$. More details can be found in the data analysis section.

Prioritising unsold birds. Vendors keep track of unsold birds. We assume that a proportion $P_{priority}$ of vendors in our simulations prioritise selling these birds before recently purchased birds. The remaining vendors, instead, do not prioritise repurposed chickens over those bought in the current day.

3 PDN setup

This section illustrates the generative algorithm responsible for instantiating the PDN. The following subsections reflect the sequential steps of the algorithm. The final goal is to generate a kind of supply chain with multiple intermediate nodes under certain structural constraints.

The algorithm first instantiates 'source' and 'sink' nodes, i.e. farms and markets, where chickens are first introduced and sold last, respectively. Then, it populates markets with vendors, compatibly with incoming bird flux as determined by farm production and region-to-market fluxes. Finally, our algorithm instantiates middlemen, i.e. the intermediate actors.

We assume here for simplicity that all actors handle and trade a single chicken breed.

3.1 Geography setup

Using external specifications about the study area, the algorithm creates a list of N_A regions, each being characterized by a given location and a weight proportional to the share $O_a^{(0)}, a = 1, \ldots, N_A$ of chickens produced there. In the case of Bangladesh we identify individual regions with upazilas. In addition, we also specify a matrix $f_{a,l}$ representing the proportion of chickens that end up in market $l = 1, \ldots, N_M$ from area a. Note that $\sum_l f_{a,l} = 1$.

3.2 Farm generation

Farm properties. In this work we consider only farms raising a single batch of chickens at a time. For this type of farm then, farm size S_F is equivalent to batch size; the latter is assumed to vary across farms and is sampled from a truncated negative binomial distribution. In addition, batch size is invariant, in the sense that a particular farm will always raise batches with the same size. The raising time τ_{raise} is assumed to be a constant and shared by all farms. Analogously to batch size, a random value of minimum rollout duration $\tau_{rollout}$ is assigned to each farm according to a probabilistic distributions but does not change over the course of a simulation. Finally, replenishment time $\tau_{replenish}$ is assumed to be sampled from a shifted negative binomial distribution, namely $\tau_{replenish} \sim 1 + \mathcal{NB}(p_{\tau_{replenish}}, n_{\tau_{replenish}})$, whenever a farm completes a rollout. Farm properties and model parameters describing farm generation are listed in Table A.

Farm locations. For each farm we draw a random location according to the following algorithm: we first draw a region a, either uniformly at random with probability P_{random}, or proportionally to the outgoing flux $O_a^{(0)}$ with complementary probability. Then, we sample a random point within the selected region. Alternatively, empirical or simulated data on farm spatial distributions could be used to fix their locations.

Farm output. Once all farms have been generated, we compute a range of quantities that are fundamental to PDN generation and/or dynamics.

First, we compute the expected daily bird output O_i from farm $i = 1, \ldots, N_F$. We compute O_i by assuming that farm i completes every rollout in exactly $\tau_{rollout,i}$ days yielding:

$$O_i = \frac{S_{f,i}}{\langle \tau_{replenish} \rangle + \tau_{rollout,i} + \tau_{raise} - 1} \,, \tag{7}$$

it should be noted, however, that this overestimates the true expected daily output O_i since $\tau_{rollout,i}$ represents only the minimum rollout duration, which might take longer in absence of middlemen to collect birds. Nonetheless, we will stick to the heuristic calculation in Eq. (7) to evaluate other quantities. These include expected daily bird output from region a:

$$O_a = \sum_{i \in a} O_i \,, \tag{8}$$

where the sum runs over all farms located in region a. Given the regional outputs, it is possible to compute the following quantities:

- $\tilde{O}_a = O_a / \sum_{a'} O_{a'}$, a normalized version of O_a.
- $q_{a,l} = O_a \cdot f_{a,l}$, the expected daily bird flux from region a to market l.
- $M_l = \sum_a q_{a,l}$, the expected daily bird flux impinging on market l.

Table A: **Farm-specific parameters.** A fraction f_{random} of farms are allocated in random upazilas, while remaining farms are assigned to upazilas proportionally to their volume of traded chickens. Farm locations are completely random within upazilas. Farm size and replenishment time distributions are $\mathcal{NB}(p, n)$. Farm size S_F is further constrained in the range $[S_F^{min}, S_F^{max}]$. Farm size is sampled once per farm per realisation: a farm always recruits the same amount of birds. Refill and rollout times are sampled during each production cycle.

Parameter	Meaning	Distribution/Value	Source
N_F	Number of farms	1200	-
f_{random}	Prob. random allocation	0.3	Assumed
T'	Farm update time	11 a.m.	-
S_F	Farm size	$\mathcal{NB}(p_{S_F}, n_{S_F})$	Estimated
p_{S_F}	Farm size $\mathcal{NB}$ parameter	0.003544	Estimated
n_{S_F}	Farm size $\mathcal{NB}$ parameter	5.245	Estimated
$S_F^{min/max}$	Min/max farm size	600, 4180	Estimated
τ_{raise}	Raising time	32 days	[1]
$p_{\tau_{replenish}}$	$\tau_{replenish}$ $\mathcal{NB}$ parameter	0.2013	Estimated
$n_{\tau_{replenish}}$	$\tau_{replenish}$ $\mathcal{NB}$ parameter	4.488	Estimated
$P(\tau_{rollout})$	Min. rollout time distribution	$(0.2, 0.3, 0.3, 0.2)$ for $\tau_{rollout} = 1 - 4$ days	Assumed

3.3 Market setup

Details about markets are provided externally. At this stage, the algorithm instantiates N_M markets, each structured in $L_{max} + 1$ empty tiers. Market properties and model parameters describing market generation are listed in Table B.

Table B: **Market parameters.**

Parameter	Meaning	Distribution/Value	Source
N_M	Number of LBMs	20	-
T_{open}	LBM opening time	6 a.m.	-
T_{close}	LBM closing time	11 p.m.	-
$G_{l,l'}$	Prob. vendor purchasing in LBMs l sells in l'	Explored	Modelled
$f_{a,l}$	Prop. of chickens leaving upazila a for LBM l	Empirical	[2]

3.4 Vendor setup

Vendor properties. At setup, each vendor is assigned a batch size S_V, drawn from a probabilistic distribution, the latter being different for W and R. Here, S_V denotes the maximum number of chickens they can purchase in a single day. Parameters describing properties of vendors and their generation are listed in Table C, while parameters relating to market tiers are listed in Table D.

Tier-by-tier vendor allocation. As explained in the main text, each vendor operates in up to two markets, and always in the same tier. Our algorithm assigns vendors to markets and tiers within them in a way that is compatible with the expected flux of chickens entering the market, as well as with between-tier fluxes. Importantly, neither the number of W and R can be specified a priori as they are determined by our algorithm, which we define as follows.

Starting from tier $L = 0$, we assign as many R and W to that tier so that their combined capacities S_V match the expected R and W incoming fluxes $M_l \cdot p_R^{(0)}$ and $M_l \cdot p_W^{(0)}$ from middlemen. Then, we assign a random destination market l' with probability $G_{l,l'}$ to each vendor generated in the current tier; If $l' = l$, a vendor will operate in a single market.

We then proceed by evaluating the expected flux of chickens $M_l^{(1)}$ impinging on tier $L = 1$ due to vendors in tier $L = 0$; this amounts to compute the combined expected output from all $L = 0$ wholesalers buying chickens in any market and selling in l. The expected wholesaler output is taken to be equal to

S_V. Given $M_l^{(1)}$, we allocate as many R and W to match fluxes $M_l^{(1)} \cdot p_R^{(1)}$ and $M_l^{(1)} \cdot p_W^{(0)}$. Finally, we repeat the same scheme for the deeper tiers as well.

As a final note, we allow for a multiplicative scaling factor $F_V^{(extra)}$, to be applied to $M_l^{(0)}$, whose effect is to allocate more vendors than what would be implied by our heuristic calculation of farm outputs. $F_V^{(extra)}$ stacks with the effects of another scaling factor $F_{MM}^{(extra)}$ (see the middlemen setup section), so that the expected flux is increased by a composite factor $F_{MM}^{(extra)} \cdot F_V^{(extra)}$. Here we set $F_V^{(extra)} = 2$ and $F_{MM}^{(extra)} = 1.4$.

Note that a vendor buying S_V chickens daily also sells the same amount of chickens on average. To see that, let us consider the following discrete-time process: during time step t, a vendor accumulates S_V chickens and sells all of his stock with probability p_{empty}, or a proportion $1 - \rho_{unsold}$ with complementary probability. The average surplus at time step $t + 1$ is given by:

$$u_{t+1} = (1 - p_{empty}) \cdot \rho_{unsold} \cdot (u_t + S_V) \, .$$

At stationariety we must have $u_{t+1} = u_t \equiv u^*$, which implies that the amounts of chickens sold and purchased must balance each other, hence our claim. Solving for u^* yields:

$$u^* = \frac{(1 - p_{empty}) \cdot \rho_{unsold} \cdot S_V}{1 - (1 - p_{empty}) \cdot \rho_{unsold}} \, ,$$

and an average daily stock, after acquiring S_V chickens:

$$S_V + u^* = \frac{S_V}{1 - (1 - p_{empty}) \cdot \rho_{unsold}} \, .$$

Finally, we take an individual's vendor maximum carrying capacity C_V as being 1.5 times the average daily stock:

$$C_V = \frac{1.5 \cdot S_V}{1 - (1 - p_{empty}) \cdot \rho_{unsold}} \, .$$

3.5 Middlemen setup

Middlemen properties. Middlemen are assumed to trade a single chicken breed. Each middleman is assigned a capacity S_{MM} from a discrete distribution, denoting the maximum number of chickens it can buy in a single day. Analogously to vendors, the number of middlemen can not be specified a priori, as it is determined dynamically: we allocate as many vendors so that their combined capacity matches the expected daily output from farms, i.e. $\sum_i O_i$. Similarly to vendors, we allow for a multiplicative scaling factor $F_{MM}^{(extra)} = 1.4$, to be applied to $\sum_i O_i$, whose effect is to allocate more middlemen than what would be implied by our heuristic calculation of farm outputs.

Table C: **Vendor-specific parameters.** W,R denote wholesalers and retailers, respectively. When some surplus is generated (with probability p_{empty}), it is sampled from a negative binomial distribution with mean $m = \rho_{unsold} \cdot n$, where n denotes total chickens offered for sale, and overdispersion parameter α_{unsold} (the variance is $m + \alpha_{unsold} * m^2$).

Parameter	Meaning	Distribution/Value	Source
S_V	Max chickens purchased	Empirical	[2]
p_{empty}	Prob. no surplus	0.32 (W), 0.15 (R)	Estimated
ρ_{unsold}	Prop. surplus	0.13 (W & R)	Estimated
α_{unsold}	Overdispersion surplus	0.29 (W & R)	Estimated
$P_{priority}$	Prop. vendors selling older chickens first	0.84 (W), 0.49 (R)	Estimated

In addition, each middleman is assigned an integer k_m, sampled from a probability distribution, representing the number of markets visited daily. The middleman will then commit to sell chickens to k_m different markets during each day (k_m does not change in time). Properties of middlemen and model parameters describing middlemen generation are listed in Table E.

Middlemen initial positions. As explained in the main text, at any point in time, each middleman tracks a set of n_{scout} distinct regions. During PDN generation, we assign middlemen to regions as follows: we first select an initial area a uniformly at random. Then, $n_{scout} - 1$ new areas are chosen among those neighboring a, i.e. within a distance $d < d_{MM}$ (based on their centroids). In case not enough areas are selected, we choose among neighbors' neighbors.

4 Simulating epidemic spread

Our simulator allows to simulate transmission of multiple pathogens/strains in the same poultry population. The current version of the simulator allows to simulate transmission across multiple scales, including at the level of the same flock and at the level of farms. All parameters describing pathogen transmission are listed in Table F.

4.1 Within-farm transmission

Let us consider a population of chickens within a single setting, e.g. a farm, a middleman's truck, a market, or a vendor's shed. Note that at any time, any chicken belongs to one and only one setting.

We simulate transmission using Sellke's construction [3]. Briefly, we assign a hazard value h to each chicken, sampled from an exponential distribution

Table D: **Market tiers' parameters.** Parameters $p^{(L)}_{W,R}$ represent the proportions of chickens sold respectively to wholesalers and retailers in tier L from either middlemen (if $L = 0$) or wholesalers from the previous tier (if $L > 0$). $1 - p^{(L)}_W - p^{(L)}_R$ represents instead the proportion of chickens that wholesalers in tier $L - 1$ ($L > 1$) sell to end-point consumers ($p^{(0)}_W + p^{(0)}_R = 1$ since middlemen do not sell to end-point consumers). Values are the same for all markets. Parameters are estimated from reconstructed transaction networks. The last tier contains only zeros as vendors in the previous tier sell only to end-point consumers.

L	$p^{(L)}_W$	$p^{(L)}_R$
0	0.606	0.394
1	0.407	0.315
2	0.169	0.318
3	0	0.058
4	0	0.

with unit rate; then, any contact with an infectious chicken reduces the target chicken's hazard by an amount δh. Whenever h hits 0 due to an infectious contact, the target chicken is infected. Later, h is updated with another draw from an exponential distribution with unit rate.

We make the assumption that chickens within the same setting mix homogeneously at random. Therefore, infectious contacts are directed at random chickens. During simulations, each infectious chicken makes exactly one contact per time step with a randomly chosen chicken from the same setting.

Infection triggers a chain of events depending on the specified compartmental model. In SIR-like dynamics, an infected chicken becomes infectious immediately, but recovers after an infectious period $\hat{t}_I$ sampled from a geometric distribution with PMF:

$$P(\hat{t}_I = k) = (1 - p_I)^k \cdot p_I \; , k = 0, 1, \dots \; , \tag{9}$$

where $p_I = 1 - exp(-(T_I + 1)^{-1})$ and T_I is the average infectious period. It is easy to check that the distribution in Eq. (9) has mean T_I. In simulations, $\hat{t}_I$ is sampled immediately after an infection happens, say time t, and recovery is deferred to time $t + \hat{t}_I$. In models with latency, i.e. SEIR-like models, chickens become infectious only after an incubation time $\hat{t}_E$ sampled from a geometric distribution with mean T_E. $\hat{t}_E$ is sampled immediately after infection at time t, and the chicken becomes infectious only at time $t + \hat{t}_E$, at which point the corresponding infectious period is also sampled.

Let us now consider an infectious chicken i trying to infect chicken j with pathogen x. The overall hazard reduction δh can then be written as:

Table E: **Middleman-specific parameters.** Number of markets serviced daily is geometric with probability p_{k_m} (truncated above by the number of markets). k_m is sampled only once per middleman per realisation.

Parameter	Meaning	Distribution/Value	Source
S_{MM}	Cargo size	Empirical	[2]
P_{move}	Daily movement prob.	0.1	Assumed
n_{scout}	Number of scouted areas	4	Assumed
d_{MM}	distance between neighboring areas	80 km	Assumed
p_{k_m}	Markets serviced parameter	0.66	[2]

$$\delta h = \beta(x,i) \cdot S(x,j) \cdot w_{setting} \;, \tag{10}$$

where $\beta(x,i)$ is the transmissibility of x and $S(x,j)$ is susceptibility of chicken j to infection with x. The factor $w_{setting}$ is a multiplier that depends only on the current setting, and accounts for differences in transmission across settings. The factors $\beta(x,i)$ and $S(x,j)$ may depend on the state of the infector and infectee, respectively. Susceptibility may account for example for previous exposure to the same pathogen, or cross-reactions induced by exposure to other pathogens/strains. In the single-strain SIR model, for example, $S(x,j) = 0$ if j is infectious or recovered.

4.2 Inter-farm transmission

We allow pathogens to spread between distinct farms. If a farm f contains infectious chickens, the probability of infecting another farm f' (provided f' is not empty), irrespective of whether f' is already infected or not, is computed as:

$$p_{f,f'} = min\{1, n_f \cdot n_{f'} \cdot K(d_{f,f'})\} \;, \tag{11}$$

where n_f, $n_{f'}$ are the average numbers of chickens in farms f and f', respectively, $d_{f,f'}$ is the distance between f and f' and $K(x)$ is a spatial kernel. If transmission occurs, a random chicken in f' is set as infected, conditional on not being already immune, and a random infector chicken is selected from farm f. If the infector is co-infected with multiple strains, a single carried strain s is chosen at random and is transmitted to the infectee. Note that this process bypasses the hazard rate calculation and that all strains are equivalent in the context of inter-farm transmission.

We simulate transmission between farms using the Conditional Entry algorithm [4]. The algorithm requires farms to be assigned to cells in a grid in order to exploit the fact that transmission is more likely to occur within cells than

between them. We use an adaptive algorithm described in the same work to construct a grid over the farm population. The algorithm relies on a hyper-parameter λ, here set to 15, that only affects the sizes and number of individual cells. Finally, because performing inter-farm transmission is computationally expensive, we run the conditional entry algorithm only once per day rather than every time step.

In this work we consider a power-law transmission kernel:

$$K(d) = \begin{cases} \beta_{FF}, & \text{for } d < d_K \\ \beta_{FF}\left(\dfrac{d_K}{d}\right)^{\gamma_K}, & \text{for } d \geq d_K \end{cases} \tag{12}$$

where β_{FF} denotes the overall strength of spatial transmission.

4.3 External introductions

External transmission events are responsible for seeding pathogens in farms. Once a day we iterate over all farms and reduce the hazard of a randomly selected chicken i due to pathogen s by an amount:

$$\delta h_{ext,i} = \beta_{ext}(s) S(x,i) \,. \tag{13}$$

In the main manuscript, we also consider an alternative seeding protocol that introduces different strains in distinct upazilas.

Table F: **Epidemic parameters.** Distributions of latent and infectious period are geometric with expected values typical of AIV infections. The transmission kernel's shape and parameters γ_K and d_K are instead inspired to a study of H5N1 epidemics in the region around Dhaka.

Parameter	Meaning	Distribution/Value	Source
T_E	Mean latent period	6 hours	[5]
T_I	Mean infectious period	48 hours	[5]
β	Base within-farm transmissibility	0.2 hours^{-1}	Assumed
β_{ext}	Introduction rate	0.0005 days^{-1}	Assumed
β_{FF}	Inter-farm transmissibility	$5 \cdot 10^{-11}$ days^{-1}	Assumed
γ_K	Exponent transmission kernel	0.94	[6]
d_K	Scale transmission kernel	0.1 km	[6]
w_X	Setting-specific transmission multiplier	0.2 (F), 1 (MM) 1 (M), 1 (V)	Assumed

References

[1] Suman Das Gupta, Md. Ahasanul Hoque, Guillaume Fournié, and Joerg Henning. Patterns of Avian Influenza A (H5) and A (H9) virus infection in backyard, commercial broiler and layer chicken farms in Bangladesh. *Transboundary and Emerging Diseases*, 68(1):137–151, 2021. _eprint: https://onlinelibrary.wiley.com/doi/pdf/10.1111/tbed.13657.

[2] Natalie Moyen, Md Ahasanul Hoque, Rashed Mahmud, Mahmudul Hasan, Sudipta Sarkar, Paritosh Kumar Biswas, Hossain Mehedi, Joerg Henning, Punam Mangtani, Meerjady Sabrina Flora, Mahmudur Rahman, Nitish C. Debnath, Mohammad Giasuddin, Tony Barnett, Dirk U. Pfeiffer, and Guillaume Fournié. Avian influenza transmission risk along live poultry trading networks in Bangladesh. *Scientific Reports*, 11(1):19962, October 2021. Number: 1 Publisher: Nature Publishing Group.

[3] Thomas Sellke. On the Asymptotic Distribution of the Size of a Stochastic Epidemic. *Journal of Applied Probability*, 20(2):390–394, 1983. Publisher: Applied Probability Trust.

[4] Stefan Sellman, Kimberly Tsao, Michael J. Tildesley, Peter Brommesson, Colleen T. Webb, Uno Wennergren, Matt J. Keeling, and Tom Lindström. Need for speed: An optimized gridding approach for spatially explicit disease simulations. *PLOS Computational Biology*, 14(4):e1006086, April 2018. Publisher: Public Library of Science.

[5] Annemarie Bouma, Ivo Claassen, Ketut Natih, Don Klinkenberg, Christl A. Donnelly, Guus Koch, and Michiel van Boven. Estimation of Transmission Parameters of H5N1 Avian Influenza Virus in Chickens. *PLOS Pathogens*, 5(1):e1000281, 2009. Publisher: Public Library of Science.

[6] Edward M. Hill, Thomas House, Madhur S. Dhingra, Wantanee Kalpravidh, Subhash Morzaria, Muzaffar G. Osmani, Mat Yamage, Xiangming Xiao, Marius Gilbert, and Michael J. Tildesley. Modelling H5N1 in Bangladesh across spatial scales: Model complexity and zoonotic transmission risk. *Epidemics*, 20:37–55, September 2017.
