# Supplementary figures and images for: EPINEST, an agent-based model to simulate epidemic dynamics in large-scale poultry production and distribution networks
Source: PLoS Comput Biol. 2024 Feb 21;20(2):e1011375. doi: 10.1371/journal.pcbi.1011375 (PMC10911595; doi:10.1371/journal.pcbi.1011375)

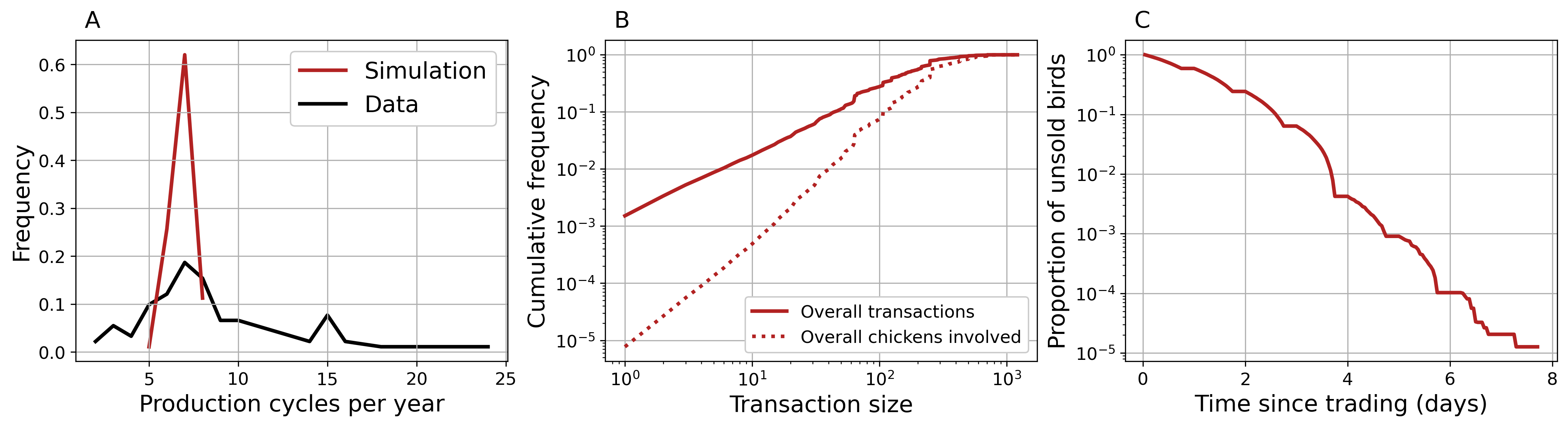

Supplement: S1 Fig — (A) Distribution of numbers of production cycles completed per year. The simulated distribution (red) appears narrower compared to empirical data (black) [27]. However, it should be added that several interviewed farmers raised multiple batches simultaneously, and those that declared raising a single batch during the interview may well have being managing 2 or more simultaneously during the previous year. (B) Cumulative distribution of sizes of transactions involving farms and middlemen (solid line). The dotted line represents the cumulative proportion of chickens sold in transactions up to a given size. The corresponding distributions, denoted with ps and ps′ respectively, are related since ps′=s·ps/∑ss·ps. In other words, ps′ is the size-biased version of ps. (C) Proportion of chickens remaining unsold after a given time since being offered for sale for the first time by a farmer. Note that it is highly unlikely for a chicken to remain unsold for more than 5 days. Results are obtained from a single simulation with default settings as in Fig 2 in the main manuscript. (PNG) [file pcbi.1011375.s002.png]

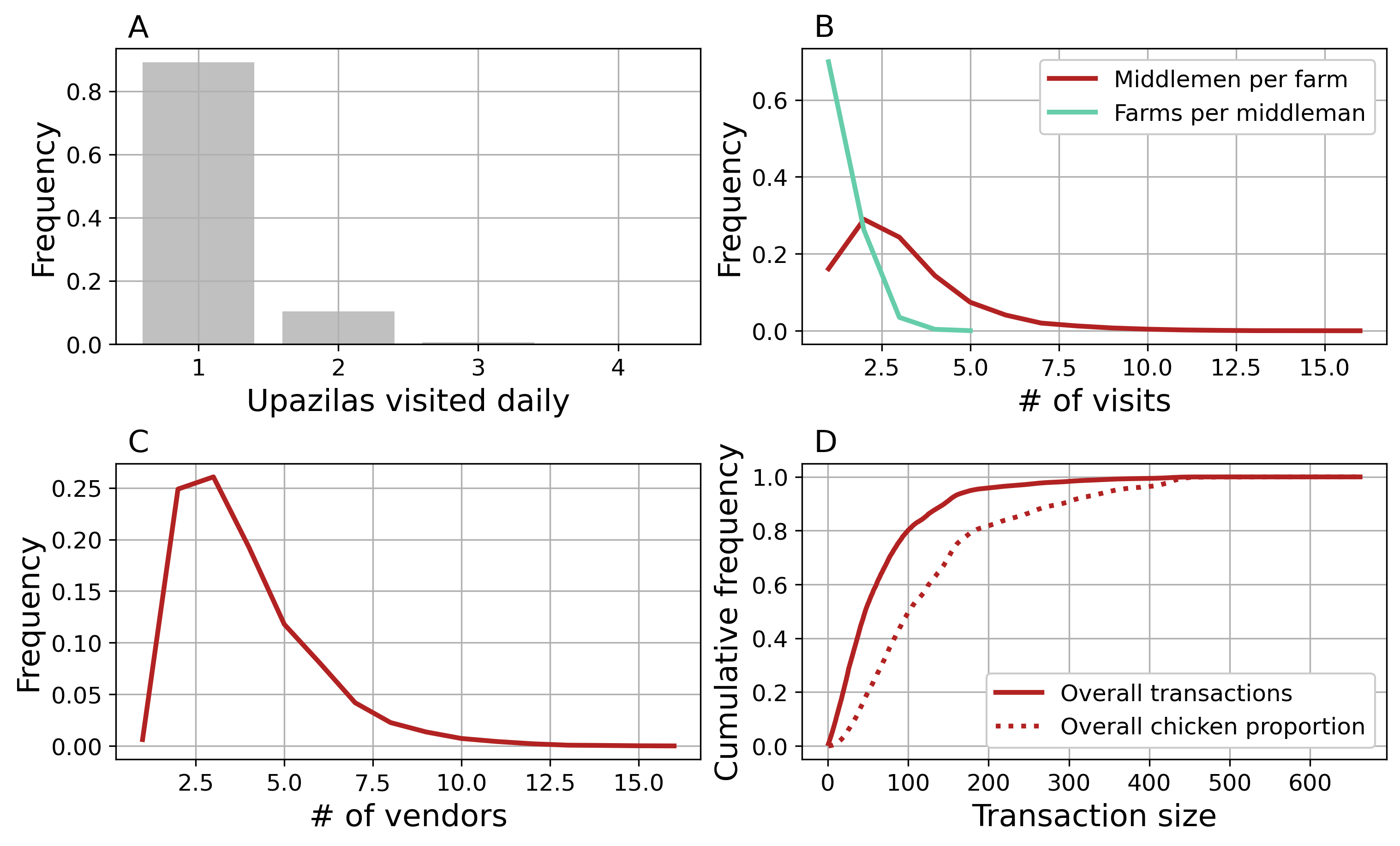

Supplement: S2 Fig — (A) Proportion of upazilas visited daily by one middleman during a single simulation. Note that a middleman may visit up to 4 upazilas per day, but visiting one or two is usually sufficient to complete a cargo. (B) Distributions of daily numbers of farms visited by one middlemen (cyan) and middlemen visiting one farm (red). (C) Distribution of numbers of vendors trading daily with a middleman. (D) Cumulative distribution of sizes of transactions involving middlemen and vendors (solid line). The dotted line represents the cumulative proportion of chickens sold in transactions up to a given size. Note that these transactions are typically smaller than those between farms and middlemen (S1 Fig) since vendors deal with smaller amounts of chickens than other PDN actors. Results are obtained from a single simulation with default settings as in Fig 2 in the main manuscript. (PNG) [file pcbi.1011375.s003.png]

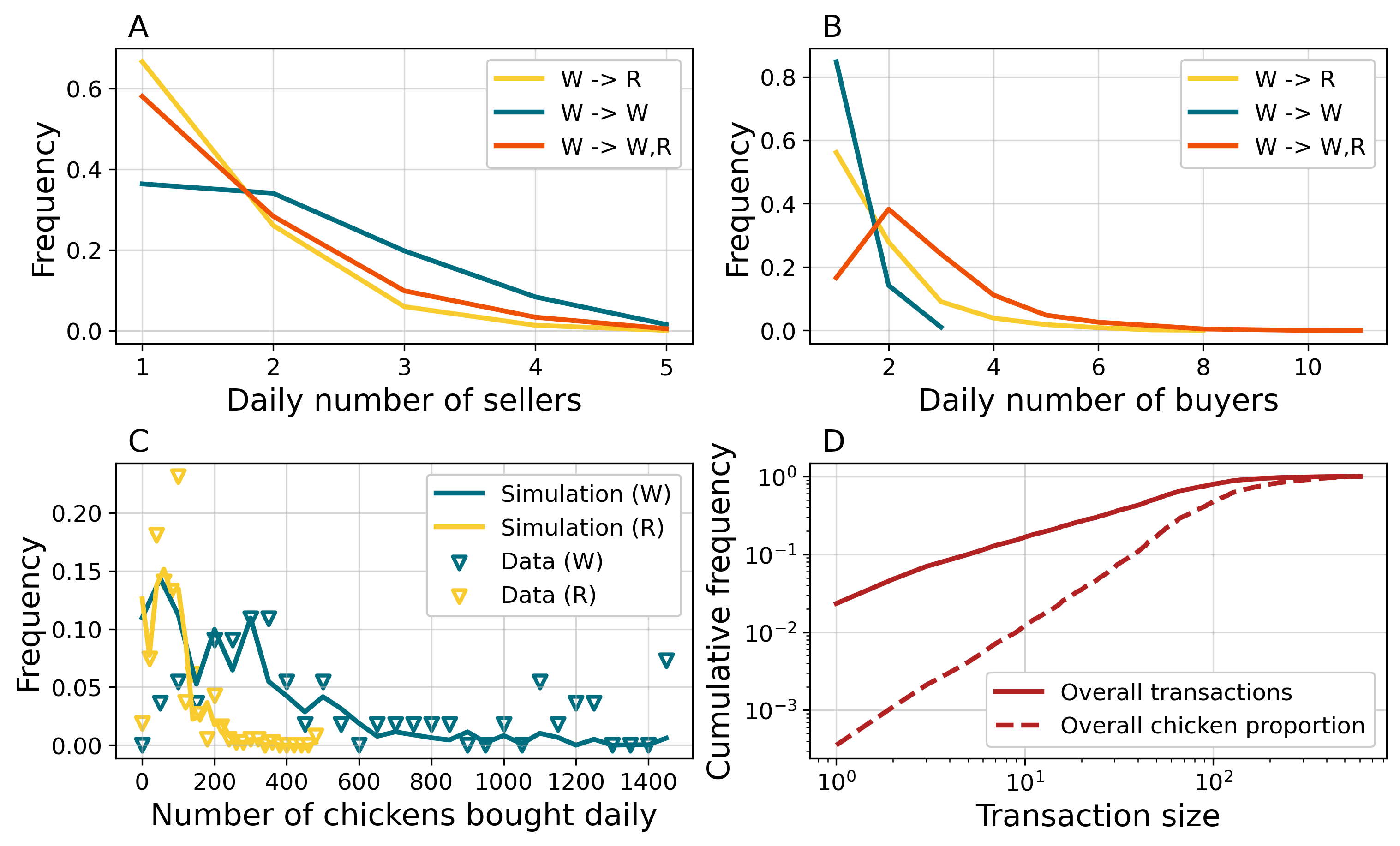

Supplement: S3 Fig — (A) Distribution of numbers of wholesalers supplying a retailer (yellow), another wholesaler (blue) or any vendor (red) on a daily basis. (B) Distribution of numbers of retailers (yellow), wholesalers (blue) or vendors (red), regardless of type, purchasing from a single wholesaler on a daily basis. Note that (A) excludes vendors buying chickens from middlemen, i.e. vendors operating in the first LBM tier. (C) Distributions of daily amounts of chickens bought from retailers (yellow) and wholesalers (blue) in simulations (lines) and data (markers) [18]. (D) Cumulative distribution of sizes of transactions involving middlemen and vendors (solid line). The dotted line represents the cumulative proportion of chickens sold in transactions up to a given size. Results are obtained from a single simulation with default settings as in Fig 2 in the main manuscript. (PNG) [file pcbi.1011375.s004.png]

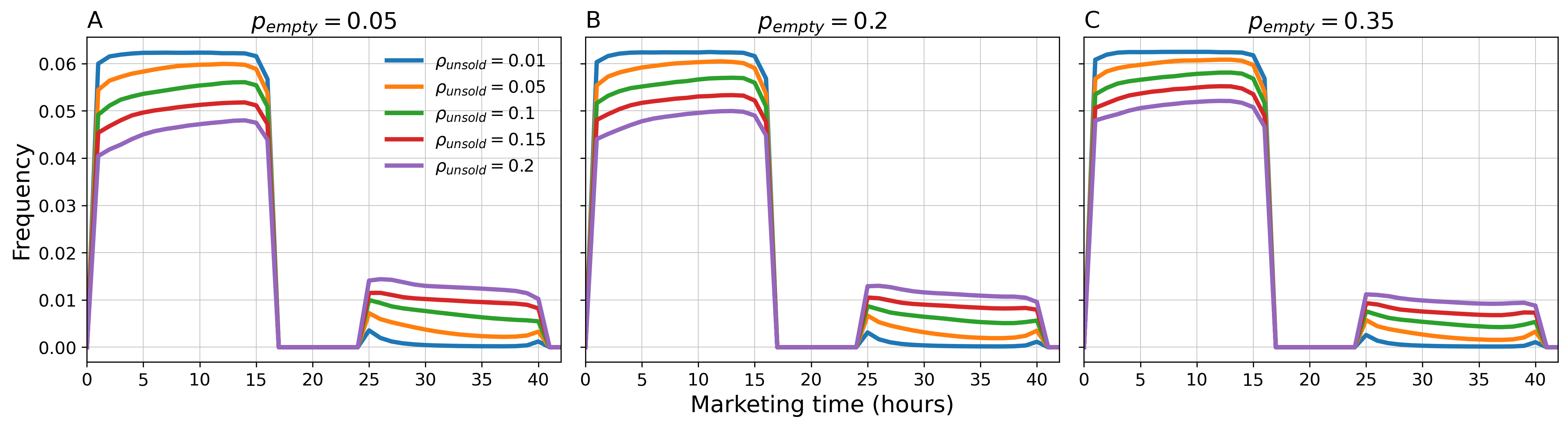

Supplement: S4 Fig — Each panel shows distributions of marketing times for different average proportions of unsold chickens ρunsold and for increasing probability pempty of a vendor selling all chickens in a single day (from left to right). The marketing time is defined as the time interval elapsed since a chicken enters any LBM for the first time and is sold to an end-point customer. Simulation settings are the same as in Fig 4 with only 10% of vendors prioritizing the sale of unsold chickens. (PNG) [file pcbi.1011375.s005.png]

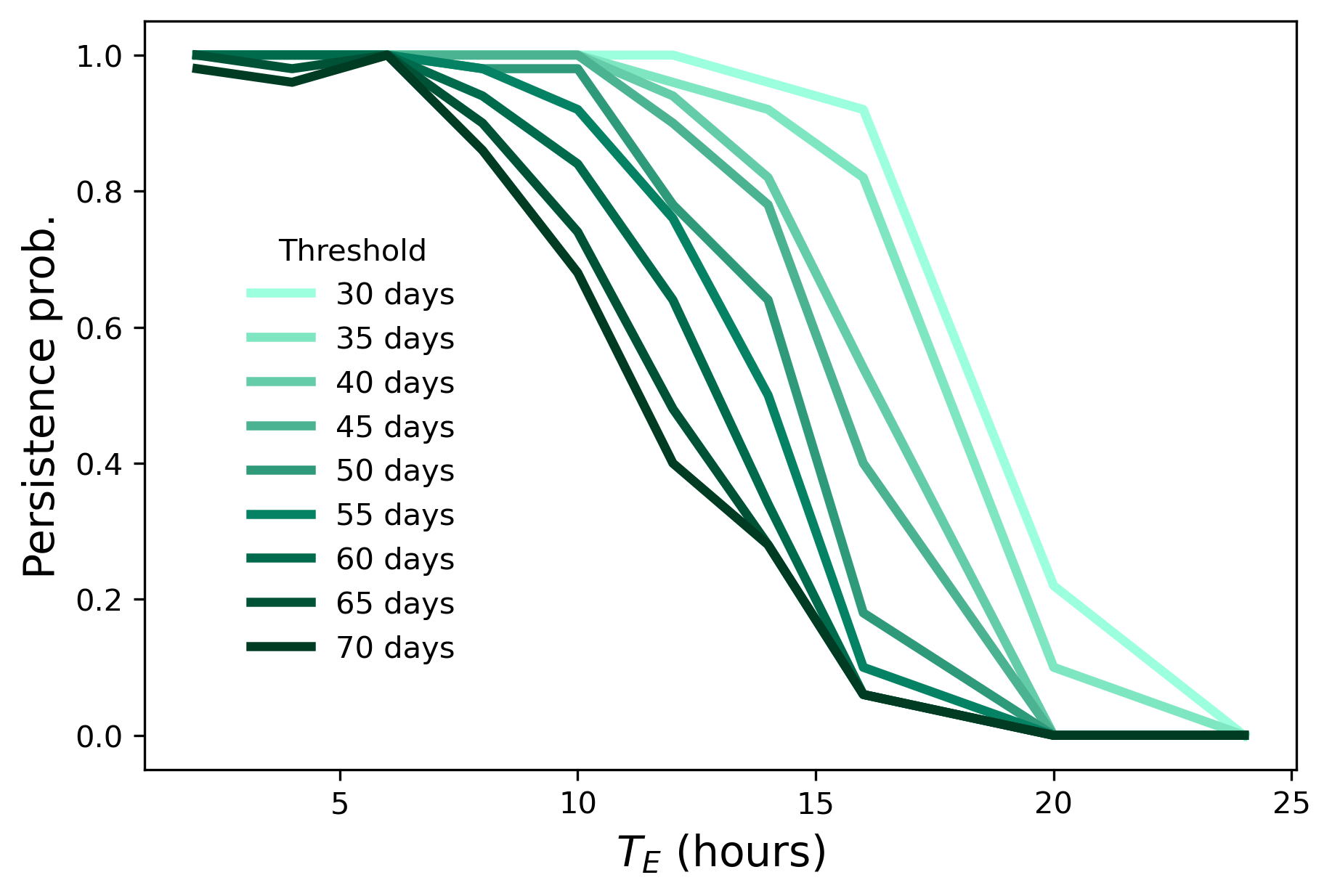

Supplement: S5 Fig — Lines show how the probability of pathogen persistence varies with both TE and the minimum duration to determine whether a transmission chain is persistent or not. The estimation of the probability of persistence as well as simulation settings are the same as in Fig 6I. (PNG) [file pcbi.1011375.s006.png]

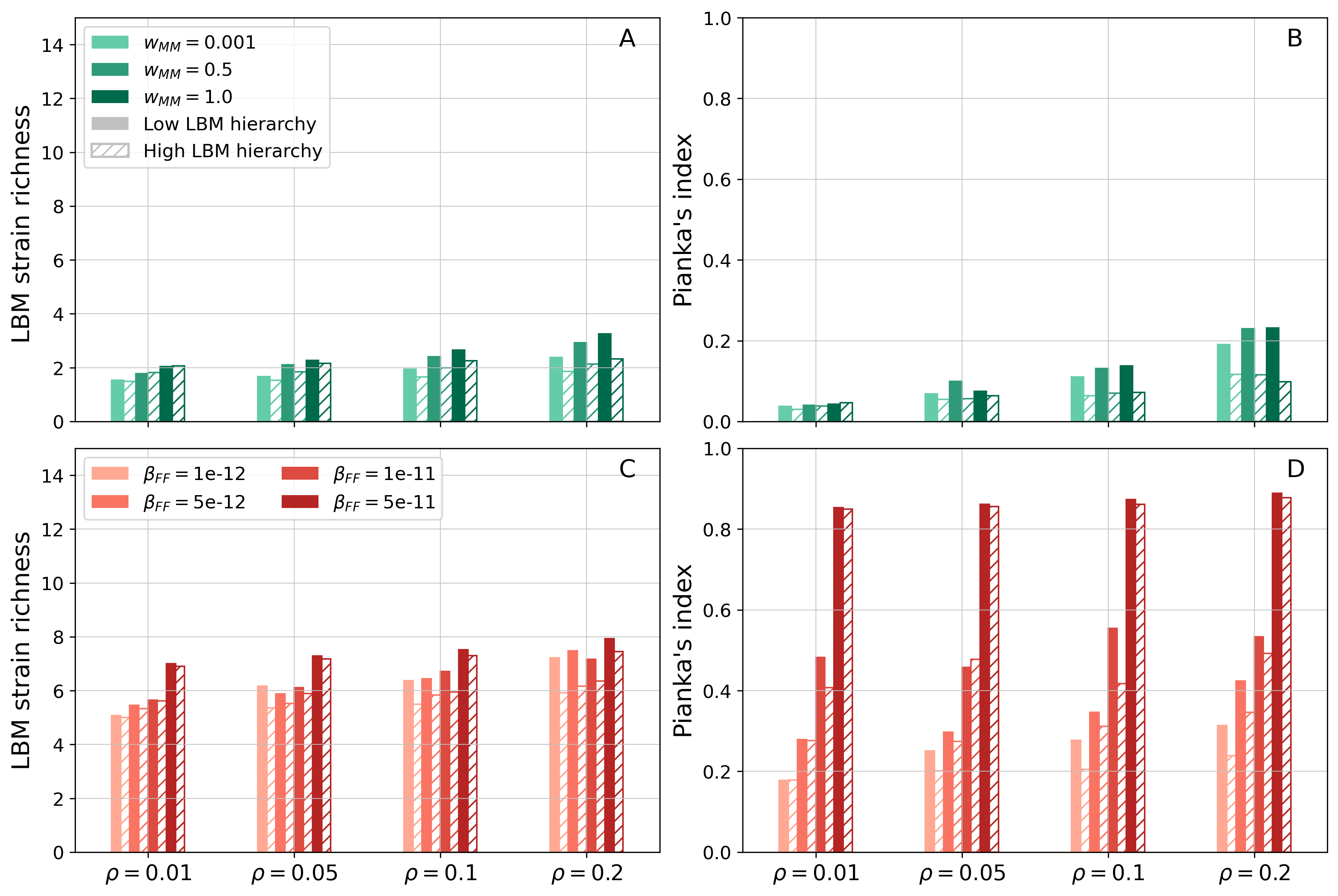

Supplement: S6 Fig — Results mirror panels B,C,E,F from Fig 7 in the main manuscript, under the assumption of complete cross-immunity (σ = 0). Increasing cross-immunity lowers strain richness in any setting as individual strains face increased competition. Nonetheless, increasing cross-immunity does not significantly affect overlap between LBMs. (PNG) [file pcbi.1011375.s007.png]

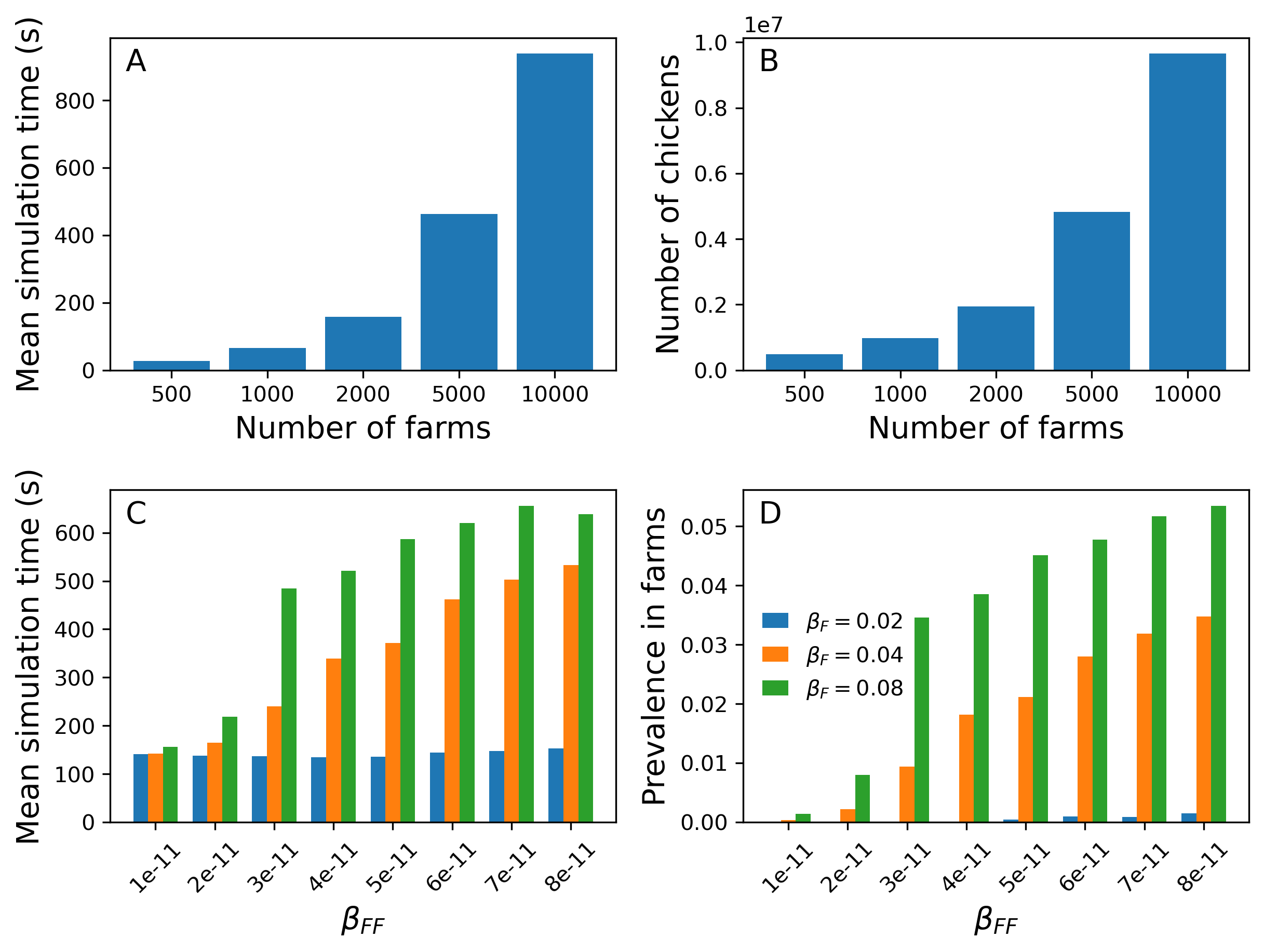

Supplement: S7 Fig — (A) Mean simulation time (seconds) as a function of the number of farms and in absence of pathogen transmission. (B) Mean size of the chicken population as the number of farms increases. (C) Mean simulation time in presence of pathogen transmission for different combinations of within- and inter-farm transmission intensity (parameters βF = β ⋅ wF and βFF, respectively). Here the number of farms is set to 1200. (D) Mean pathogen prevalence in farms (proportion of infected farmed chickens) as a function of the same parameters. Our findings indicate that simulation time increases linearly with the size of the poultry population and non-linearly with pathogen prevalence when parameters βF and βFF are varied. This happens because large values of prevalence can be achieved only if the pathogen is able to transmit sufficiently well both within and across poultry flocks. Results are averaged over 50 simulations from 5 different PDN realisations. In panels A,B, each simulation is run for 6 years. In panels C,D, each simulation is run for 8 years and the pathogen is introduced after 4 years. For simplicity, we consider transmission within settings other than farms to be negligible (we set wMM = wM = wV = 0.001). Other PDN and epidemiological parameters are set to default values. (PNG) [file pcbi.1011375.s008.png]
