# Supplementary material for: EPINEST, an agent-based model to simulate epidemic dynamics in large-scale poultry production and distribution networks
Source: PLoS Comput Biol. 2024 Feb 21;20(2):e1011375. doi: 10.1371/journal.pcbi.1011375 (PMC10911595; doi:10.1371/journal.pcbi.1011375)
Supplement: Attachment — Submitted filename: point_by_point_reply.pdf [file pcbi.1011375.s009.pdf]

Notes for reviewers: we provide a version of the revised manuscript and supplementary methods with salient changes highlighted in red/blue. New text is highlighted in blue, while removed text is highlighted in red and is striked through.

Reviewer #1

The authors present a very well written, well documented paper with an elaborate study of the effect of poultry farming and trading practices on pathogen spread, maintenance and evolution. The model is well explained in the main document and documented in detail in the supplementary information, so it is relatively easy for the reader to find model assumptions and parameter values. My comments are minor and given below.

We are pleased that the reviewer appreciated our manuscript and we thank them for their positive assessment of our work.

General remarks:
• It would be good to mention in the abstract, introduction and also the title that this work concerns broilers, as the epidemic dynamics in layer hens would be very different.

The reviewer raises a good point, in that we tailored applications to a broiler farming system. This was a deliberate choice as we were mainly concerned with introducing the agent-based model through relevant case studies. However, this framework could be easily tailored to other production systems by changing, e.g., the distribution of farms and their sizes, rearing times, and including the possibility to have overlapping production cycles (i.e. multiple batches of different ages being raised at the same time). We hence believe that, as stated elsewhere in the manuscript, this tool holds potential to describe poultry production systems other than broiler ones. In our opinion, the current title adequately conveys this belief, but we agree that the abstract lacks specificity about the production system to which the model is applied in this manuscript. The latter now explicitly mentions that applications in this work are tailored to broilers. The corresponding sentence now reads:

"We provide example applications to broiler production in Bangladesh, but the modular structure of the model allows for easy parameterization to suit specific countries and system configurations"

• In supplementary material, line 464 it is mentioned that "We make the assumption that chickens within the same setting mix homogeneously at random". This is a valid choice for the modelling, but does not necessarily reflect reality, especially in very large farms. Can you spend some attention on this modelling choice in the discussion and explain in what ways the model could deviate from reality because of this choice.

We agree with the important point raised by the reviewer. We now discuss this modelling choice in the discussion:

"An important assumption in EPINEST is that chickens mix homogeneously within a given setting. This simplification may be less accurate for larger farms that store multiple flocks in separate sheds, or for large villages where multiple backyard poultry flocks are raised. Heterogeneity in local transmission could be partly recovered by 'splitting' large farms in smaller, neighbouring units and by exploiting inter-farm transmission (as if different units were distinct farms)."

• Chapter "Epidemic dynamics", line 197. At the LBMs, are infectious (I) chickens removed from the population, or are they allowed to recover (R)? I can't imagine that people want to buy sick, infected chickens.

We confirm that infectious chickens are allowed to recover in our simulations, even when offered for sale in LBMs. The reviewer is right in pointing out that manifest signs of sickness may make sick chickens less palatable to customers (Fearnley & Zhang, Preventive Veterinary Medicine, 2023). While not stated explicitly in the manuscript, epidemic simulations neglect disease severity and are arguably more appropriate to describe the dynamics of a low-pathogenic AIV strain (we only wrote that we considered an AIV-like pathogen that does not kill infected chickens). Our modelling assumptions were heavily influenced by our experience with H9N2 AIV, a low-pathogenic strain that is zoonotic and highly prevalent in LBMs and causes only mild to subclinical symptoms in chickens. In this context, we would expect only a minority of infected chickens to be sick enough to affect customer choices, thus justifying our assumptions as a first approximation.

Following this comment, we have now better contextualised our modelling assumptions in the introductory paragraph in the "Epidemic dynamics" Results subsection. The text now reads:

"Importantly, we assume that infected chickens do not die due to the disease and that sick chickens are not removed from LBM stalls. This assumption is broadly compatible with the epidemiology of low-pathogenic AIV strains such as H9N2, which are highly prevalent in Bangladeshi LBMs and only cause mild to sub-clinical symptoms in chickens."

Detailed remarks
• Figure 2, panel B.
○ The text mentions that black lines show expected values, while red lines are for measured values. The legend on the other hand assigns red to simulations and black to data. Which is correct?

The "expected" distribution indeed refers to the black line (the data), which we strived to reproduce, albeit approximately (please note that we are not explicitly enforcing the distribution of trading phase duration in the model), in simulations. We agree that this wording is confusing. We have therefore changed this sentence in the caption to improve clarity.

○ Explain the abbreviation PMF in the figure legend. The legend should be readable on its own. Please check other figures too (include CMF).

We decided to replace PMF and CMF within individual figures with "frequency" and "cumulative frequency" to improve readability.

○ How was the fit of the red lines assessed?

Fig. 2B, and similarly panels C and G, only compare realised distributions (red lines) of certain statistics of poultry movements with their empirical counterparts. No formal assessment was carried out here as the objective of this figure was to illustrate the ability of the model to generate realistic poultry movements according to a range of summary statistics. While the match between simulations and some observations is not perfect, we stress that we did not enforce all of the statistics shown here. Trading rollout duration (B), the number of visits per batch (C) and the length of stay of chickens in the market (F), for example, are emergent properties of PDN dynamics. There are other statistics, however, that are enforced more tightly in simulations, as is the case for the number of visits to the market (E) or relative fluxes of chickens between individual upazilas and markets (D). Indeed, these panels show a better match between simulations and data. We have emphasised this point in the caption of Fig. 2:

"We emphasize that some of the quantities shown here (panels B,C,G), emerge dynamically during simulations and are not enforced as tightly as poultry fluxes (D) and visits to LBMs (E)."

• Figure 7. " (D-G) Simulations with inter-farm..." should be " (D-F) Simulations with inter-farm...".

This has now been fixed.

Reviewer #2

The article is well written and the work done is impressive . Just few very minor points that could improve the impact of the paper
The first time $\beta_{FF} is introduced no definition is given. I would suggest to make a Table (maybe in the Supplementary Information) collecting all the parameters used , their definition and their estimates or sources

We thank the reviewer for their very positive assessment of our manuscript.

Indeed, β_{FF} was not defined in the main text. We have now amended this by providing a short description of inter-farm transmission in the results section, at the point where β_{FF} is introduced for the first time. We added a further reference to Table F in the Supplementary Text S1, that summarises epidemiological parameters used.

"In the model, the intensity of inter-farm transmission is proportional to a spatial kernel $K(d)$ decaying with spatial distance d and such that $K(0) = \beta_{FF}$. The latter parameter thus represents the maximum extent of inter-farm transmission at $d = 0$ (see also S1_Text and Table F therein)."

A second , maybe more important point, wold be to provide a table resuming the performances of the tool: comparing different scenarios, size of populations , network structures and sizes,and the time to run a set of simulations. This could be done as supplementary material

We thank the reviewer for their suggestion. We measured the performance of the tool within a range of scenarios. First, we considered a scenario with no pathogen transmission, i.e. we simulated only poultry movements, and varied the number of farms from 500 to 10000. We find that simulation time scales linearly with the latter, which drives the size of the poultry population (middlemen, LBMs and vendors hold much smaller numbers of chickens at any point in time). Second, we set the number of farms to 1200 and ran epidemic simulations under varying levels of within- and between-farm transmission through parameters w_F and β_{FF}, respectively. Figure S7 now reports $\beta_F = \beta \cdot w_F$ rather than w_F. We made this choice to assess the impact of pathogen prevalence on simulation time. Changing β_{FF} affects the number of infected farms and hence the number of outbreaks; also, the inter-farm transmission process can be particularly time-consuming depending on how many farms are infected. Our findings indicate that simulation time increases with pathogen prevalence but the dependence on w_F and β_{FF} is non-linear: large values of prevalence can be achieved only if the pathogen is able to transmit sufficiently well both within and across poultry flocks. These results are summarised in supplementary Fig. 7, which is also reported below.

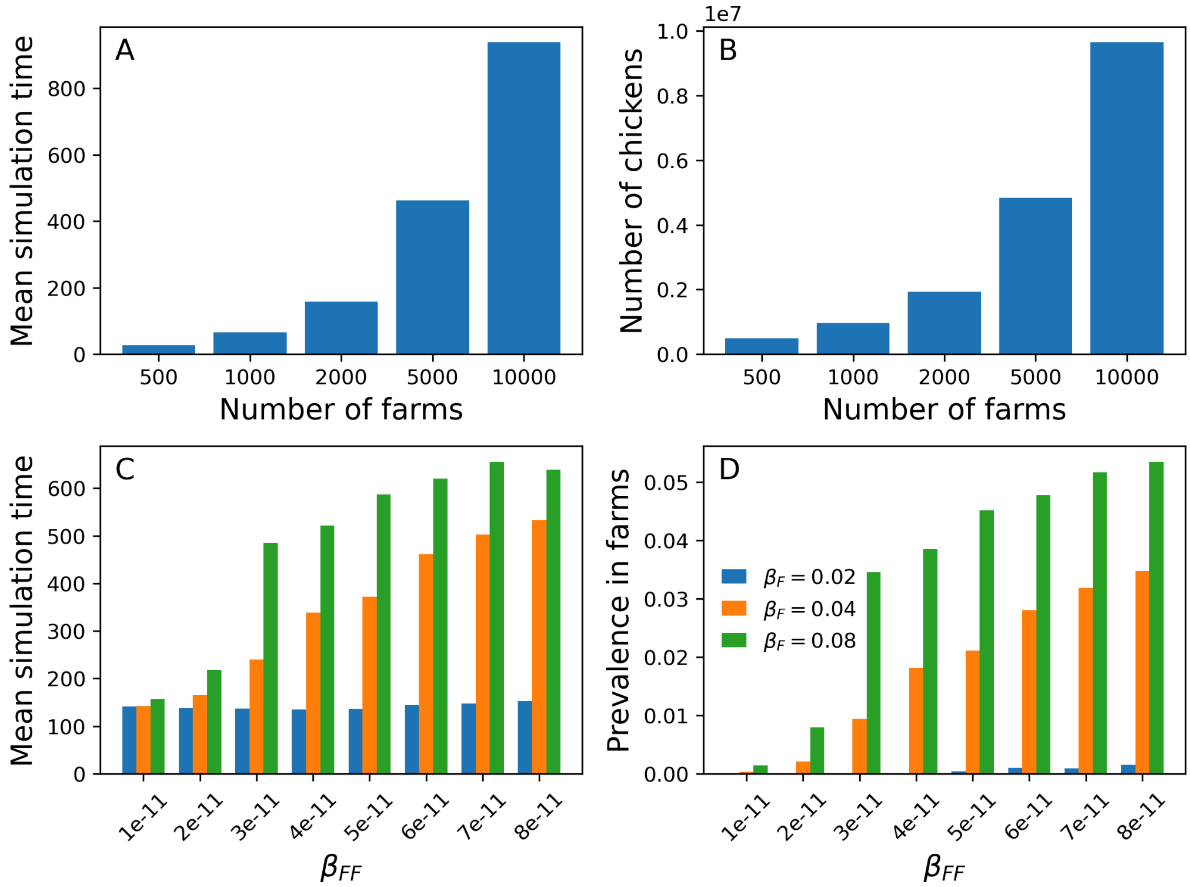

Supplementary Figure 7. **Simulation time analysis.** (A) Mean simulation time (seconds) as a function of the number of farms and in absence of pathogen transmission. (B) Mean size of the chicken population as the number of farms increases. (C) Mean simulation time in presence of pathogen transmission for different combinations of within- and inter-farm transmission intensity (parameters $\beta_F = \beta \cdot w_F$ and β_{FF}, respectively). Here the number of farms is set to 1200. (D) Mean pathogen prevalence in farms (proportion of infected farmed chickens) as a function of the same parameters. Our findings indicate that simulation time increases linearly with the size of the poultry population and non-linearly with pathogen prevalence when parameters β_F and β_{FF} are varied. This happens because large values of prevalence can be achieved only if the pathogen is able to transmit sufficiently well both within and across poultry flocks. Results are averaged over 50 simulations from 5 different PDN realisations. In panels A,B, each simulation is run for 6 years. In panels C,D, each simulation is run for 8 years and the pathogen is introduced after 4 years. For simplicity, we consider transmission within settings other than farms to be negligible (we set $w_{MM} = w_M = w_V = 0.001$). Other PDN and epidemiological parameters are set to default values.

Third, I would suggest to change the color scale used in Figure 6 D_I,

We appreciate the reviewer's suggestion, but we prefer to keep the current colour scale.

Reviewer #3

1. The article represents a significant model development effort and describes a novel ABM approach that enables capturing the complexities of disease transmission dynamics in poultry production and distribution networks. The modeling framework incorporates flexibility to evaluate specific PDN related questions informed by data as well as more open-ended questions and sensitivity analysis. In addition, the framework

enables assessing the impact of network structure aspects (e.g., hierarchy in vendor transactions), mixing and pathogen amplification along the value chain on the disease transmission dynamics in live bird markets. The capability to model multiple co-circulating strains and strain diversity is a unique feature relative to existing models.

We thank the reviewer for their positive assessment of our manuscript.

2. The current PDN modeling scenario does not consider the impact of environmental contamination in LBMs and potential farm exposures via vehicles or personnel returning from LBMS towards disease persistence and strain richness. I suggest discussing these as potential limitations as applicable.

The reviewer is right in pointing out that environmental contamination in LBMs and potential farm exposures via vehicles or personnel returning from LBMs can affect the pathogen transmission and persistence.

We discussed these limitations in the Discussion:

"In its current implementation, EPINEST assumes pathogens to spread directly from infected to susceptible chickens. However, environmental contamination, caused by infected chickens, can also affect transmission and contribute to pathogen dispersal within and between sites, especially from LBMs back to farms via contaminated mobile traders. Future versions of this software may incorporate these additional routes of transmission."

3. Are there any recommendations for potential mitigations at various levels of the PDN to reduce AI persistence, prevalence and strain mixing at LBM based on the simulation results?

We thank the reviewer for this comment. Our objective here was to present the model rather than making a thorough investigation of AIV transmission, which would be required to produce informed recommendations. We have added a new paragraph in the Discussion about the possibilities offered by our framework to assess the effectiveness of interventions:

"This ABM framework would allow users to conduct a more comprehensive assessment of intervention effectiveness, thereby yielding more meaningful recommendations. For interventions classically implemented in other disease transmission models such as culling and vaccination, for example, the ABM enables the evaluation of their impact across the entire PDN. Moreover, the model facilitates the exploration of interventions that seek to modify the PDN configuration—such as rewiring the network of mobile traders and/or vendors—an aspect that has been overlooked in the modelling literature despite its relevance for mitigating disease risks."

4. Line 10: typo such instead of “suck risk”

This has now been fixed.

5. Line 262: LBMs. “In contrast, a larger degree of hierarchy in movements (striped bars) had the opposite effect, in agreement with findings from Fig. 5.” As the impact of hierarchy is a key insight, suggest elaborating this statement to improve clarity (i.e., impact of hierarchy on network reachability and overlap potentially contributing to reduced strain richness)

We have expanded this sentence, which now reads:

"In contrast, a more hierarchical layout of vendor movements decreased strain richness and overlap across LBMs. This is consistent with findings from Fig.\:\ref{Fig_market}, which are suggestive of random inter-market connections promoting chicken's access to multiple LBMs. These results highlight the importance of accounting for inter-market movements to analyse the mixing of co-circulating AIVs."

6. Line 418: Define δi,j used in equation 1 and how was it was estimated. Also, could you provide further explanation on how δi,j is positive when the outdegree ki is 0 and thus there are no connections between markets I and J?

We thank the reviewer for spotting the missing definition of $\delta_{i,j}$. The latter denotes Kronecker delta, which takes value 1 if $i = j$ and is 0 otherwise. Therefore, $k_i = 0$ implies $G_{ii} = 1$ and $G_{ij} = 0$ for $j \neq i$, consistently with the absence of connections emanating from i. We have now defined $\delta_{i,j}$ in the main text.

7. Lines 414-418: Is the outdegree for each LBM informed by data or is it calculated and updated dynamically in the generative network model? Consider expanding this paragraph to explain the network growth mechanism in greater detail.

As the reviewer correctly suggest, the outdegree k_i is updated dynamically during the growth process, which adds nodes and edges one at a time. The growth mechanism simply provides a recipe to generate network topologies with varying density of connections and hierarchy. We have now expanded this paragraph to improve clarity.

8. Probability that a vendor purchases in LBM l and sells in l’ is referred to as wl,l’ in the supplement (Table A2) and Gij in the main text (line 410). Are these two variables equivalent? If not, could you clarify the difference.

The reviewer is right: these variables are indeed equivalent. We replaced $w_{l,l'}$ with $G_{l,l'}$ everywhere throughout the manuscript and supplementary material.

9. Line 22 in supplement: Why was a negative binomial distribution chosen for the replenishment time. Were any other statistical distributions explored?

We fitted both a Poisson and a negative binomial distribution to empirical replenishment times from farms that declared to use a single shed (see also panel R in Figure A in the S1 Text). We did not perform a proper model selection exercise, but noted that the negative binomial fit was visually better than the Poisson fit at capturing overdispersion in empirical times. This is now mentioned in S1 text, which also refers to panel R in the paragraph discussing replenishment times.

10. Supplement Table A6: How was the value of βFF (5 * 10−11 days−1) chosen in the baseline scenario?

This value was chosen through trial and error in order to yield a significant amount of inter-flock transmission for illustrative reasons (Fig.6B). In real applications, β_{FF} should be calibrated using data, e.g., on numbers of infected farms.

11. Supplement equation 12 and Table A6: Is the transmission kernel K(D) in the same form as in reference 6? If not provide details on how the parameters γK and Dk were obtained from the reference.

We confirm that we used the same kernel function as in the reference mentioned above. d_K was set to 0.1 km as in that reference, while the exponent γ_K was obtained from their Table S5 (parameter α, "Wave 2" column, $d_K = 1 + \alpha$).
